# Age-Related Changes to Eating and Swallowing Impact Frailty: Aspiration, Choking Risk, Modified Food Texture and Autonomy of Choice

**DOI:** 10.3390/geriatrics3040069

**Published:** 2018-10-12

**Authors:** Julie A. Y. Cichero

**Affiliations:** School of Pharmacy, Pharmacy Australia Centre of Excellence, The University of Queensland, 20 Cornwall Street, Brisbane QLD 4102, Australia; j.cichero@uq.edu.au; Tel.: +61-7-3446-1900

**Keywords:** dysphagia, aspiration, choking, pneumonia, sarcopenia, texture modified food, ethical decision making

## Abstract

Reductions in muscle mass and strength are well known complications of advancing age. All muscles of the body are affected, including those critical to chewing and swallowing. A diagnosis of frailty and its features of weakness and unintentional weight loss are particularly relevant to the aging swallowing system. Age related changes to eating and swallowing function means that there is a natural tendency for elders to self-select ‘soft’ foods due to loss of dentition and fatigue on chewing. However, it is not well known that tooth loss and poor dental status is associated with increased choking risk, especially as people age. In fact, people over 65 years of age have seven times higher risk for choking on food than children aged 1–4 years of age. Texture modified foods are provided clinically to reduce choking risk and manage dysphagia. Although certain food textures offer greater swallowing safety, they significantly restrict food choice. This commentary paper will highlight age-related changes to the eating and swallowing system, noting especially those that are relevant for frail elders. Swallowing impairments also affect the ability to manage liquids, and aspiration risk in healthy and frail elders is also discussed. Modified food textures that are most often recommended by clinicians to maintain sufficient oral intake and reduce choking risk will be described, while also highlighting the nutritional challenges associated with these foods and offering some solutions. The ethical challenges associated with balancing the autonomy of choice of food textures with swallowing safety will be addressed.

## 1. Introduction

In seeking to foster healthy aging, the World Health Organisation has called for identification and prevention of frailty. A physical frailty syndrome is defined when three or more of the following five specific criteria are present [1,2]:

1. Weakness measured by low hand grip strength
2. Slowness measured by decreased walking speed
3. Low level of physical activity
4. Low energy or self-reported exhaustion
5. Unintentional weight loss

While there is a gradual decrease in physiologic reserve with normal aging, this is more marked in individuals with frailty, resulting in a striking change in health state or difficulty recovering from seemingly minor events such as infection, surgery or changes in medication [3]. In Fried’s landmark study of individuals over the age of 65 years, 5% were identified as frail and 47% as pre-frail. Subsequent studies have noted that the risk of frailty increases with age such that 26% of individuals over the age of 85 years are identified as frail [3]. Difficulty swallowing, or dysphagia, has been identified both as a contributing factor to frailty and a consequence of it [4]. Well known complications of dysphagia include malnutrition, dehydration and susceptibility to pneumonia. These conditions will contribute to weakness, low energy and unintentional weight loss. The prevalence of dysphagia increases with advancing age such that 10–20% of individuals older than 65 years are estimated to have swallowing difficulties [5]. However, the prevalence of other comorbidities such as stroke, dementia and Parkinson’s Disease also increase with advancing age. Hence it is difficult to tease out pure age-related risks associated with dysphagia from those associated with comorbidities that so often accompany aging.

During the normal aging process, it is recognised that there are reductions in muscle mass, fatty infiltration of muscle and reduction in muscle strength and that these contribute to frailty [6]. Muscle atrophy of the respiratory, skeletal and swallowing systems has recently been reported [7]. In order to accommodate changed function associated with aging elders alter their food texture choices to those that are ‘easier to chew’, ceasing to eat certain foods and inadvertently contributing to weight loss [8]. In addition, food texture modification is often used in the treatment of dysphagia to encourage sufficient intake where chewing strength is reduced and also to reduce choking risk [9,10]. The trade-off for improved ease of chewing and swallowing is a reduction in visual appeal, nutrient density and restricted food choices [11,12]. These elements provide ethical challenges to balance autonomy of food choice with unintentional weight loss [13]. In order to best address the impact of age-related changes to eating and swallowing on frail elders without pathologic co-morbidities, it is important to (a) identify at-risk individuals and (b) ensure there is adequate quality and quantity of food to limit weight loss. This review addresses normal age-related changes to eating and swallowing and aspiration and choking risks for frail elders, helping to identify at-risk individuals. It concludes with information on food texture modification and its impact on food choice and the ability to ensure adequate quantity and quality of food to limit weight loss. It is recognized that weight loss is a key risk factor for frailty.

## 2. Age-Related Changes to Eating and Swallowing

As we age there are a number of anatomical and physiological changes that affect eating and swallowing function. For example, ossification of the hyoid bone and thyroid and cricoid cartilages, atrophy of intrinsic laryngeal muscles, dehydration of the laryngeal mucosa, loss of elasticity of laryngeal ligaments and flaccidity and bowing of the vocal folds are noted with aging [14]. These changes affect the extent and duration of hyolaryngeal excursion and laryngeal vestibule closure. Whilst chewing function remains intact, reductions in muscle strength and loss of teeth impact food choices, chewing efficiency and post swallow oral residue. Reductions in tongue driving force and mild delays in triggering the swallow reflex increase pharyngeal residue contributing to an increased rate of clearing swallows but no associated increase in penetration or aspiration [15]. The duration of laryngeal excursion and closure is retained until about 60 years of age and then declines [16]. The general slowing of the aging eating and swallowing system mimics age related slowness seen in other systems, such as gait and mobility. Sensory changes are also apparent such as a reduction in olfaction appreciation (smell) and loss of taste. Daily medication use so common in old age, is also known to negatively impact sensation of taste and olfaction. Chemisthesis appreciation, however, is retained with a sustained ability to detect the heat in chilli, or the coolness of menthol [17].

Normal aging sees a decline in food intake and this is known as the anorexia of aging [18]. The fundus of the stomach is less compliant, allowing a greater filling of the stomach antrum that triggers signals to the central nervous system to cease eating [18]. Concurrently there are also changes to the response to, or amount of circulating hormones such as cholecystokinin, leptin and testosterone that impact satiety signals and contribute to reduced food intake. Changes to appreciation of taste and smell may reduce hunger drive while tooth loss may impact food texture choices [17,18]. A reduction in sensory-specific satiety is also noted from 65 years of age such that elders are less inclined to seek novel food and instead choose to eat the same food day after day [17].

A decrease in food intake may predispose to vulnerability for muscle wasting. Where there is a significant loss of muscle mass and function, it is known as sarcopenia; this is a hallmark feature of frailty [19]. Whilst sarcopenia is more commonly associated with appendicular skeletal muscles for walking and hand grip, muscle wasting is also seen in the muscles associated with eating and swallowing. There is a cross-sectional reduction in jaw muscles associated with aging and change in muscle fibre composition. Korfage [20] notes that the proportion of pure Type I (‘slow twitch’) masseter fibres decrease with age while the proportion of pure Type II (‘fast twitch’) masseter fibres increase. Muscle fibre changes are also noted in the aging diaphragm where there is atrophy of Type II muscles. These changes reduce the effectiveness of expulsive airway behaviours, such as coughing and sneezing, providing a mechanism that increases potential risk for development of pneumonia [21]. 

Fatty infiltration of skeletal muscle also causes weakness by reducing the physical integrity of the muscle and is also a marker of aging and frailty [6]. As an analogy, changes to the integrity of muscle architecture could be likened to the slow yet devastating damage done to wooden structures invaded by white ants. The outside of the structure still looks sound, however, due to critical loss of wood the structure is unstable and susceptible to collapse with little provocation. Age related fatty infiltration has been found in muscles relevant to eating and swallowing. For example, fatty infiltration has been noted in the geniohyoid muscle that has a role in anterior and superior movement of the hyoid during swallowing [22]. Although the authors had hypothesised that fat infiltration of the geniohyoid would be associated with increased aspiration risk, this was not supported by their study.

High levels of sarcopenia are associated with decreased tongue strength [23]. A reduction in tongue strength has been associated with an increased risk for aspiration as it increases the likelihood of bolus retention in the pharynx [24]. Of interest, Butler et al., also found there to be a moderate correlation between reduced hand grip strength and reduced posterior tongue strength [24]. Although more research is required, reductions in hand grip strength may alert clinicians to potential tongue weakness [4].

## 3. Aspiration Risk: Healthy and Frail Elders

With structural changes to muscles used for chewing and swallowing with aging and a loss of functional reserve, it is not surprising to find that a diagnosis of frailty is associated with impaired swallowing safety. A study of frail elderly patients showed that more than two thirds presented with oropharyngeal residue, more than half presented with laryngeal penetration of the bolus and 17% demonstrated tracheobronchial aspiration. Impaired tongue propulsion and delayed hyolaryngeal excursion was linked to oropharyngeal residue across liquid thickness levels. These features were not evident in healthy controls [25]. As noted above, changes in muscle integrity associated with sarcopenia could be contributing to the reduced tongue propulsion and delayed hyolaryngeal excursion noted in these elders. At one year follow up, mortality rates were significantly higher in frail elderly patients (56% vs. 15%) with impaired swallow efficiency or safety. 

A systematic review and meta-analysis of frail elderly indicates that dysphagia is a significant risk factor for the development of aspiration pneumonia with an OR of 9.84 (95% CI 4.15–23.33) [26]. Aspiration pneumonia most often develops due to micro-aspiration of saliva, or bacteria carried on food and liquids, in combination with impaired host immune function [27]. Recent studies have demonstrated that chronic inflammation of the lungs is a key feature in aspiration pneumonia in frail elderly nursing home residents [28]. The authors describe cases of frail elderly who show sporadic fever (one day per week for several months). On radiology review of these individuals there is evidence of chronic inflammation in the consolidated lung tissue, linking chronic micro-aspiration and chronic lung inflammation. Thus, Ebihara and colleagues recommend recognising chronic inflammation as opposed to acute inflammation as a precursor to the development of aspiration pneumonia [28]. The cough reflex is a critical safeguard against the development of pneumonia. Ebihara and colleagues demonstrated that even if material is aspirated, pneumonia is less likely to develop if the aspirated material is completely expelled by coughing. Thus, a reduced coughing ability appears to increase the risk of developing pneumonia and should be identified as a risk factor in frail individuals.

The cough reflex has motor, sensory and cognitive components [28]. Although there is no apparent decrease in cough reflex *sensitivity* in either active or frail elderly, the ‘urge to cough’ decreases with normal aging and is severely reduced in the frail elderly, even with the strongest stimuli. A cortical and neurological overlay has been proposed as the reason for reduced ‘urge to cough’ in elders. As noted above, the inability to clear aspirated material from the lungs is one of the mechanisms by which pneumonia may develop. Once aspiration pneumonia has taken hold, animal and human studies find the condition induces muscle atrophy in the respiratory, skeletal and swallowing subsystems, further contributing to a downward spiral of muscle strength and worsening sarcopenia [7]. There are three important learnings from this. Firstly, that clinical efforts should actively identify individuals with poor cough function, especially if there is chronic lung inflammation. Secondly, that treatment should focus on improving coughing function in elders and especially frail elders to expel aspirated material. Thirdly, that pneumonia physically changes muscle architecture of the respiratory, skeletal and swallowing subsystems, reducing their capacity to function. To this last point, while increased energy intake is needed for individuals recovering from pneumonia, frail elders may not have the functional reserve to meet these needs without food fortification or nutritional supplementation. Where dysphagia for liquids exists, provision of high energy, high protein foods and fortification of usual meals with cream and butter are cost effective ways to provide the nutritional building blocks necessary for muscle recovery [29]. 

## 4. Choking Risk: Healthy and Frail Elders

Although dysphagia is most often associated with difficulty managing liquids, dysphagia is also relevant where there is difficulty managing solids. Frail elders have been shown to have ingestive problems associated with both eating and drinking [30]. Reviewed at mealtimes, difficulties associated with functional chewing, bolus formation and positioning were noted in frail as opposed to robust elders. As noted above, with aging there may be loss of dentition and alterations to the strength of muscles required for chewing and bolus formation. An improperly chewed and especially large solid bolus has the potential to fatally occlude the airway [31,32]. It is sobering to note that after falls, choking on food presents as the second highest cause of preventable death in aged care [33]. Although we commonly associate food choking risk with young children, data shows that individuals over 65 years of age have a choking incidence that is seven times higher than children aged 1–4 years, with men at higher risk than women [34]. Of special relevance, a diagnosis of pneumonitis is positively correlated with increased risks associated with choking on food. Poor dentition and the use of sedative and antipsychotic medications further increase risk for food choking in adults [34].

Choking risk can be viewed as it relates to food features, person features and environment features. Food features that increase choking risk include the physical textural, size and shape of foods. For example, foods that are fibrous, hard, firm, stringy, chewy, sticky, dry, crumbly, crunchy or shaped in such a way that they can occlude the airway (round or long) pose a choking risk [35]. Foods that are consistently associated with choking and reported on autopsy findings include meat, bread, sandwiches and toast, amongst others. These foods share a commonality of complex fibrous structure that is broken down most effectively by rotary chewing using molar teeth. Sufficient stamina is also needed to sufficiently prepare the bolus for swallowing, with bite-sized pieces of meat and bread requiring more than 20 chewing strokes per bolus [36,37].

Person features that increase choking risk relate to inadequate dentition, difficulty maintaining posture and positioning, fatigue during meals and impaired function as a result of medication and poor decision-making capacity. A loss of dentition affects healthy and frail elders. Although obvious, adequate dentition is critical to effective chewing and bolus preparation to prevent choking. There is a high correlation between absent teeth, ill-fitting dentures, dental disease and sudden choking deaths [32,38]. Insufficient teeth or poor fitting dentures reduce chewing efficiency to produce a coarse, poorly chewed bolus, resulting in coughing and choking [39]. The lack of adequate dentition in and of itself therefore produces an oral stage dysphagia, resulting in poor bolus preparation and formation. If poor bolus preparation is accompanied by cognitive decline that impairs the ability to decide whether the bolus has been adequately chewed, or a physical inability to spit out an improperly chewed bolus, choking risk increases.

In Fried’s definition of frailty, weakness and low energy or self-reported exhaustion are noted [2]. While frail elders may present with slow walking speed or weak hand grip strength, slowness and weakness may also be evident in static activities. For example, a review of ingestive skills at meal times demonstrated that frail elders have significantly more difficulty maintaining postural stability and adequate head position during meals. Frail elders are also significantly less likely to tolerate the physical effort of a meal compared with robust elders [30] demonstrating another person-centred choking risk factor.

The need for medication with sedative qualities, such as anti-psychotics, opioid analgesics, hypnotics and anti-anxiety medications impact the central nervous system and can impair the integrity of the swallow and cough reflexes [40,41]. Medications such as these may be prescribed for frail elders, who suffer a significant fall owing to postural instability [3]. Impairments in swallow and cough function may be evident for as long as the medication is needed. Temporary changes to food texture and increased supervision or assistance at meal times may be required for as long as the medication is needed, to reduce choking risk.

The need for supervision is an environmental risk factor for choking. As noted above, a requirement for sedating medication may increase choking risks that could be mitigated with supervision or assistance at meal times. In pathologic aging, cognitive impairment such as that which accompanies dementia, increase choking risk with behaviours such as: eating too fast, not chewing very much, over-filling the mouth, or swallowing large mouthfuls [42]. Inadequate meal time supervision of individuals at risk of choking has had fatal consequences. The provision of better mealtime monitoring was one of the key findings of a systematic review of deaths in nursing homes [9].

Features that might help identify elders at risk of eating or swallowing problems, as noted in Section 3 and Section 4, have been collated and are summarized in Table 1. In line with WHO directives, early identification allows for early preventive strategies to be put in place [1].

## 5. Food Texture Modification Used for Therapeutic Purposes

Speech pathologists offer a range of treatment options to address dysphagia, including safe swallowing strategies, postural adjustments and rehabilitation exercises. Texture modification, although a cornerstone feature of dysphagia rehabilitation, should be paired with strategies, postural adjustment and exercises wherever possible for optimal outcomes. Careful assessment by a speech pathologist provides optimal person-specific management strategies. In order to reduce choking risk for older adults, food textures are modified so that they require less oral processing. Depending on the degree of chewing impairment, or lack of dentition, food may be provided with little modification such as tender foods that can be cut with the side of a fork, to minced foods that require little chewing, to pureed foods for individuals with significant oral impairment. Texture modified foods may be prescribed to reduce choking risk, and/or with the aim of increasing food intake by combatting chewing fatigue associated with eating regular food textures. Where a high choking risk for regular textures has been ascertained, dysphagia diets recommend providing food in specified ‘bite-sized’ pieces (e.g., 1.5 cm × 1.5 cm) such that the pieces will generally not occlude the airway if accidentally swallowed whole or with minimal chewing [9,10]. 

While food texture modifications may help reduce choking risk, the texture modification process can reduce the nutrient density of the food, further exacerbating malnutrition [11]. For example, to puree a meal requires the addition of liquid so that the food is not sticky. The addition of liquid, especially water, washes out nutrient density. Flavour release that would normally occur with chewing is also impaired with texture modified food, reducing its sensory appeal. Furthermore, texture modified foods are frequently visually uninviting, often presented as ice cream ball shaped mounds of food. When foods look and taste unappealing, naturally consumption declines. It is little wonder then that studies show that individuals who require texture modified food, frequently do not meet their energy requirements [12,13]. A simple assessment of appetite has been found to be predictive of weight loss. Using four simple questions regarding (a) strength of appetite, (b) fullness when eating, (c) how food tastes and (d) how many meals the person eats a day can reliably predict weight loss in community dwelling and long-term care residents [45].

Elders in general and frail elders in particular, can little afford weight loss. Weight loss and low body weight are associated with increased risk for the development of pressure ulcers and a higher likelihood of non-healing wounds [46]. Protein lost through exudate in non-healing wounds increases protein requirement needs. As individuals advance over 70 years of age, their protein needs naturally increase by about 25%. For men, there is an increase from 0.84 g/kg/day to 1.07 g/kg/day, while for women the increase is from 0.75 g/kg/day to 0.964 g/kg/day [47]. It is easy to see that the sarcopenia cycle can flourish exponentially if not addressed quickly.

## 6. Balancing Food Texture Requirements and Autonomy of Choice with Swallowing Safety

Eating and drinking are some of the most basic of human needs according to Maslow’s hierarchy. Food is linked with mood, emotion and cultural identity and impacts social participation at mealtimes [48]. It is not surprising then that conflict and ethical dilemmas arise with the provision of texture modified food and thickened liquids. At the same time as wanting to ensure safety from preventable choking or aspiration risk, health professionals must also balance the person’s right of choice. Kaiser et al., eloquently describe that for health professionals the desire to respect autonomy of choice is often coupled with feelings of negative duty of care [49]. Thankfully ethical issues around the provision of texture modified food and thickened liquids can be successfully addressed using a shared decision-making algorithm. Repeated education and feedback regarding elements of compliance and reasons for non-compliance are important for the individual management of risk. Where non-compliance continues in the face of assumed or real risk, the health care team may alter diet modification, reinforce safety precautions, share responsibility with family and explain consequences of non-compliance. Aged care facilities provide a unique challenge. Where staff to resident ratio is low, clinicians may choose to prescribe foods that are more texture modified to allow individuals to eat safely unsupervised. Anecdotally some aged care facilities allow individuals to receive non-texture modified foods if provided and strictly supervised by a family member, similar to the case reported by Kenny [48]. Alternatively, clinicians may provide patient-centred care with specific menu item selection or alternate cooking strategies to provide food less likely to result in choking. Innovation in ways of providing texture modified food may increase compliance, address safety concerns and help meet critical nutritional targets.

To provide sufficient nutrition to keep weight stable, or to promote recovery from chronic lung inflammation, pressure ulcers, urinary tract infections or similar ailments commonly associated with aging, innovation in cooking techniques, ingredient selection and food texture modifications are needed. Cooking techniques that provide tender meats that require minimal chewing provide an option to increase protein intake. In a study of edentulous individuals, 61% prepared their food so that they could eat it as ‘very soft’ and half cut the food into small pieces to help aid eating [8]. Where texture modification requires food to be minced into small particles or pureed, the use of natural flavours such as oyster sauce, garlic and ginger may be beneficial as these have been associated with an increase in energy intake of up to 25% of hospitalised elders [50]. Likewise, inclusion of herbs or foods that promote flavour in addition to sensations such as warmth, for example oregano, thyme, black pepper or capsaicin, could be used to promote sensory appreciation using the intact trigeminal system of elders [51]. Elders naturally adjust hard food textures in order that they can still enjoy them describing dunking bread into soup or dunking hard biscuits into tea to soften them prior to eating [8]. For individuals with dysphagia, where choking risk is a concern, these natural adaptations may be possible if there is adequate staffing to provide help if needed. Textures known as ‘transitional solids’ that are initially dry and crunchy but ‘melt or dissolve in the mouth’ with moisture and require little chewing may also be of benefit [10,52]. Foods such as wafers, potato crisps or prawn crisps fall into this category. Carefully prepared moderate protein spreads such as paté or humus could be paired with transitional solids as a vehicle for increased protein intake that is socially acceptable and yet easy to chew and swallow.

## 7. Conclusions

This review paper demonstrates that age related changes in eating and swallowing function can predispose frail elders to increased risks associated with aspiration, choking and malnutrition. Reductions in hand grip strength, one of the markers of frailty, has been correlated with reductions in tongue strength and dysphagia, although further research in this area is needed. Loss of teeth or poor dental status affect the ability to chew food adequately, increasing choking risk and restricting food choices. Restrictions to food choices likely contributes to unintentional weight loss. Reductions in ‘urge to cough’ associated with aging allow for consistent micro-aspiration of saliva that may result in chronic lung inflammation and predisposition to development of pneumonia. Sarcopenic changes to the respiratory muscles reduces the ability to cough and clear material from the lungs, contributing to low energy and low physical activity that are also markers of frailty. Frail elders are likely to experience meal time difficulties including postural instability and fatigue during meals. Careful choice of food textures and ingredients is required to maximise oral intake, especially protein intake, to maintain adequate nutrition.

## Figures and Tables

**Table 1:** Summary of features that may help identify elders at risk of eating or swallowing problems.

Feature That Increases Risk of Eating or Swallowing Problems in Elders	Impact And Evidence of Increased Risk Associated With Individual Features
Increased age: Aged more than 65 years	Increased Risk of Choking on Food [34] Increased Likelihood of Dysphagia Diagnosis [4] Increased Diagnosis of Frailty [3]
Poor dental status: Dental disease, missing teeth, poorly fitting dentures	Increased risk of choking on food [32,38,39]
Postural instability during meals	Difficulty maintaining postural stability during meals more likely in frail than robust elders [30] Difficulty maintaining head position during meals more likely in frail than robust elders [30]
Poor mobility	Bedfast, increased likelihood to develop aspiration pneumonia [4,43]
Fatigue during meals	Reduced ability to tolerate the physical effort of a meal more likely in frail elders than robust elders [30]
Sedative, opioid or antipsychotic medication	Sedative, opioid or antipsychotic types of medication affect the effectiveness of cough and swallowing reflexes and have been associated with increased choking risk [34,40,41] Individuals older than 85 years take a larger proportion of medications that affect level of consciousness or swallowing response [44]
Chronic vs. Acute lung infection	Fever one day per week for several months associated with lung infection increases likelihood of developing aspiration pneumonia [28]
Reduced hand grip strength	Weak hand grip strength more than x2 likely to develop dysphagia, although further research required [4,24]
